# 氟化共价有机聚合物固相微萃取-高效液相色谱测定水产品中丁香酚类麻醉剂

**DOI:** 10.3724/SP.J.1123.2021.06027

**Published:** 2021-09-08

**Authors:** Xingyi WANG, Yanlong CHEN, Gongke LI

**Affiliations:** 1.中山大学化学学院, 广东 广州 510275; 1. School of Chemistry, Sun Yat-sen University, Guangzhou 510275, China; 2.兴义民族师范学院生物与化学学院, 贵州 兴义 562400; 2. School of Biology and Chemistry, Xingyi Normal University for Nationalities, Xingyi 562400, China

**Keywords:** 高效液相色谱, 固相微萃取, 丁香酚, 水产品, 氟化共价有机聚合物, high performance liquid chromatography (HPLC), solid phase microextraction (SPME), eugenol, aquatic products, fluorinated covalent organic polymer (F-COP)

## Abstract

氟化共价有机聚合物(F-COP)具有较大的比表面积和吸附容量,对丁香酚类化合物具有特异性吸附。该文以2,3,5,6-四氟对二苯甲醛(TFA)和1,3,5-三(4-氨苯基)苯(TAPB)为单体,三氟甲磺酸钪(Sc(OTf)_3_)为催化剂在室温下快速合成F-COP,并将其作为固相微萃取(SPME)吸附剂,结合高效液相色谱-紫外检测法(HPLC-UV),建立了测定水产品中丁香酚、乙酸丁香酚酯和甲基丁香酚麻醉剂的分析方法。通过傅里叶红外光谱、X射线衍射、N_2_吸附-解吸等温线和扫描电子显微镜等手段对F-COP材料进行表征。考察了萃取时间、搅拌速度、解吸溶剂及解吸时间对丁香酚类麻醉剂萃取量的影响,在萃取时间为30 min、搅拌速度为700 r/min、解吸溶剂为乙腈、解吸时间为10 min时,丁香酚类麻醉剂获得了最佳的萃取效果。在Diamonsil Plus C_18_-B色谱柱(250 mm×4.6 mm, 5 μm)上,以甲醇-水(60:40, v/v)为流动相,流速0.800 mL/min,进样量20.0 μL,紫外检测波长280 nm,柱温30 ℃条件下,丁香酚和乙酸丁香酚酯在10~1000 μg/L,甲基丁香酚在10~1500 μg/L范围内呈现出良好的线性关系,相关系数(*r*^2^)大于0.9961,方法检出限为2.9~4.5 μg/kg(*S/N*=3),精密度小于8.7%(*n*=5)。最后,将该分析方法用于罗非鱼和基围虾样品的3种麻醉剂残留分析中,得到了满意的回收率(76.7%~104%)。结果表明,F-COP-SPME-HPLC-UV可满足水产品中丁香酚类麻醉剂的分析检测。

## Abstract (en)

Fluorinated covalent organic polymers (F-COPs) constitute a new class of porous materials with a topological structure, large surface area, and potential superiority over other types of polymers in sample preparation. In this study, a F-COP was rapidly synthesized by a simple Schiff-based reaction using 2,3,5,6-tetrafluoroterephthalaldehyde (TFA) and 1,3,5-tris(4-aminophenyl)benzene (TAPB) as monomers, and by adding scandium (Ⅲ) triflate (Sc(OTf)_3_) as the metal catalyst at room temperature. The prepared F-COP was applied as a coating adsorbent for solid phase microextraction (SPME) to enrich three kinds of eugenol anesthetics in aquatic products. The extraction performance of an enrichment medium is an important factor for practical application in real analytical projects. This F-COP adsorbent with rich *π*-stacking electrons contained abundant phenyl rings and imine (-C=N) groups throughout the molecular framework. The adsorption mechanism was explored and discussed based on the *π-π* affinity and hydrogen bonding interaction, which contributed to its strong recognition affinity to targets. The F-COP was characterized by Fourier transform infrared (FT-IR) spectroscopy, X-ray diffraction (XRD), nitrogen adsorption-desorption isotherms, and scanning electron microscopy (SEM). The results indicated that the novel F-COP-SPME bar exhibited a rough and porous surface structure, good preparation reproducibility, and high stability. High performance liquid chromatography (HPLC) was performed with an ultraviolet-visible (UV-vis) wavelength detector. A Diamonsil plus C18 column (250 mm×4.6 mm, 5 μm) was used as the analytical column. The mobile phase comprised 60% methanol and 40% ultrapure water, and was flowed at 0.800 mL/min. The injected volume of the sample was 20.0 μL. The column temperature was maintained at 30 ℃ and the detection wavelength was set to 280 nm. Further, the SPME conditions (including extraction time, stirring rate, desorption solvent, and desorption time) that influenced the extraction efficiencies of the eugenol anesthetics were investigated in detail. Thus, the optimized F-COP-SPME bar conditions were established as follows: extraction time: 30 min; stirring rate: 700 r/min; desorption solvent: acetonitrile; desorption time: 10 min. By combining F-COP-based SPME with HPLC-UV analysis, an effective method was developed for the extraction and determination of eugenol, eugenyl acetate, and methyl eugenol residues in aquatic products. The method demonstrated good linearity in the range of 10-1000 μg/L for eugenol and eugenyl acetate, and 10-1500 μg/L for methyl eugenol, with correlation coefficients (*r*^2^) greater than 0.9961, low limits of detection (2.9-4.5 μg/kg, *S/N*=3), and excellent precision (relative standard deviations lower than 8.7%, *n*=5). Finally, the method was applied for the effective extraction of three kinds of eugenol anesthetics from tilapia and shrimp samples. The obtained recoveries were in the range of 76.7%-98.7% and 80.3%-104% with relative standard deviations of 8.5%-11.8% and 8.6%-12.4% (*n*=5), respectively. These results demonstrated that the F-COP is promising for use as an adsorbent in SPME for the determination of eugenol anesthetics in aquatic products. The developed method was suitable for the qualitative and quantitative determination of three kinds of eugenol anesthetics in aquatic products, yielding a satisfactory purification effect and sensitivity.

丁香酚作为一种渔用麻醉剂,在水产品长途运输中,可降低呼吸和代谢强度,减少碰撞,降低其死亡率而被广泛使用^[1]^。但有研究表明,高剂量的丁香酚会引起心律失常、肾脏损伤、消化系统等问题,对人类健康造成潜在危害^[2]^,因此日本食品安全法规定丁香酚在水产品体内的最大残留量为50 μg/kg^[3]^,但我国还未对其使用和残留量制定相关法规,针对其在水产品中的痕量残留检测的文献报道较少。目前,丁香酚类麻醉剂常用的检测方法有气相色谱-质谱(GC-MS)^[4,5,6,7]^、高效液相色谱-质谱(HPLC-MS)^[8]^、高效液相色谱-紫外(HPLC-UV)^[9,10]^和电化学(EC)^[11]^等,但水产品中丁香酚类麻醉剂含量少,基质复杂,对其进行准确检测存在一定困难。高效的样品前处理方法是获得准确结果的有效方法,现有液液萃取(LLE)^[9]^、固相萃取(SPE)^[4]^、分散固相萃取(DSPE)^[8]^和固相微萃取(SPME)^[7,12]^等方法应用在水产品前处理中,其中LLE方法操作简单,但很难消除水产品中色素、脂肪和蛋白质等杂质对测定的干扰,DSPE方法在处理过程中容易造成目标物损失导致回收率偏低,所以SPE和SPME技术在水产品前处理中更为常用,特别是针对水产品中一些挥发性和痕量物质检测时,SPME技术因其高效低耗、绿色环保显示出更大的优势而被广泛使用^[13]^。

SPME涂层是决定方法选择性、灵敏度、寿命、重现性和应用价值的关键。SPME涂层的种类有限,其萃取容量或选择性难以满足不同性质复杂样品的痕量分析要求,亟待发展新型SPME涂层^[14]^。氟化共价有机聚合物(fluorinated covalent organic polymer, F-COP)是一类具有拓扑结构的新型多孔聚合材料,主要由轻质原子通过较强的共价键相互连接而成,具有物理化学性质稳定、吸附容量高、孔结构和尺寸可控等特点,而且F-COP结构中含有氟官能团,可以与酚羟基之间形成氢键相互作用,从而实现对目标物的特异性识别与吸附,因此F-COP吸附剂在丁香酚类化合物的富集与分析中有很大的应用潜力^[15,16,17]^。

本文以三氟甲磺酸钪为催化剂,在室温下合成一种F-COP材料,并采用黏合法在石英棒表面制备SPME涂层,结合HPLC-UV建立了测定丁香酚、乙酸丁香酚酯和甲基丁香酚的分析方法,并将该方法成功应用到罗非鱼和基围虾的分析中,为水产品中丁香酚类麻醉剂的残留检测提供技术支持。

## 1 实验部分

### 1.1 仪器和试剂

LC-2010岛津液相色谱仪(日本岛津); H1850台式高速离心机(湖南湘仪); DZF-6020真空干燥箱(上海新苗医疗器械制造有限公司); GZX-9146鼓风干燥箱(上海博迅实业有限公司医疗设备厂); DF-101S恒温加热搅拌器(巩义予华仪器有限责任公司); KQ-300DE型超声波清洗器(昆山市超声仪器有限公司); Nicolet Magna 750傅里叶变换红外光谱仪(美国Nicolet); Gemini SEM 500场发射扫描电子显微镜(德国Zeiss); D8 Advance X射线衍射仪(德国Bruker); ASAP 2020全自动气体吸附仪(美国Micromeritics)。

2,3,5,6-四氟对二苯甲醛(TFA, 纯度98%)、1,3,5-三(4-氨苯基)苯(TAPB, 纯度97%)、三氟甲磺酸钪(Sc(OTf)_3_, 纯度99%)(毕得医药);丁香酚(纯度98%)、乙酸丁香酚酯(纯度98%)、甲基丁香酚(纯度98%)的标准品、1,4-二氧六环(无水级,纯度99.5%)(北京百灵网科技有限公司); 1,3,5-三甲苯(无水级,纯度97%)(阿拉丁试剂(上海)有限公司);聚丙烯腈(PAN,平均相对分子质量150000)(分析纯,美国Sigma-Aldrich公司); *N*,*N*-二甲基甲酰胺(DMF)、氢氧化钠、盐酸、正己烷、乙酸乙酯、四氢呋喃(分析纯,天津市大茂化学试剂厂);甲醇、乙醇、乙腈和丙酮(色谱纯,Dikma公司);石英棒(外径920 μm,纯度99.99%)(富友石英制品厂);实验中所用超纯水均为Milli-Q制备。

### 1.2 色谱条件

色谱柱:Diamonsil Plus C_18_-B(250 mm×4.6 mm, 5 μm);紫外检测波长:280 nm;流动相:甲醇-水(60:40, v/v);流速:0.800 mL/min;进样量:20.0 μL;柱温:30 ℃。

### 1.3 标准溶液的配制

准确称取10.0 mg(精确至0.2 mg)丁香酚、乙酸丁香酚酯和甲基丁香酚标准品,用色谱纯甲醇配制成400 mg/L的混合标准储备液,于4 ℃下冷藏保存备用。实验所需不同浓度溶液均用超纯水进行稀释。

### 1.4 F-COP-SPME石英棒的制备

1.4.1 F-COP材料的制备

根据文献^[18,19]^报道的合成方法并进行适当修改,制备F-COP材料。具体合成方法如下:称取TAPB (36 mg)和TFA (31 mg),加入4 mL的1,4-二氧六环-1,3,5-三甲苯(4:1, v/v)混合溶液,超声至完全溶解。在超声条件下缓慢加入2 mg Sc(OTf)_3_催化剂,室温下密封静置反应10 min,得到黄色固体物质,分别用1,4-二氧六环和甲醇超声洗涤3次(3×10 mL),然后离心分离,获得的材料在60 ℃真空条件下干燥12 h备用。

1.4.2 F-COP-SPME石英棒的制备

截取5 cm石英棒,依次用1 mol/L氢氧化钠和1 mol/L盐酸溶液各浸泡5 h,再用超纯水超声清洗后于100 ℃下烘干备用。采用黏合法制备F-COP-SPME石英棒,具体过程如下^[12]^: (a)分别称取90 mg F-COP粉末和90 mg PAN粉末于3 mL玻璃小瓶中,加入1.5 mL DMF,放入小磁子搅拌,超声分散形成均匀浆液;(b)将石英棒插入浆液中,再从浆液中缓慢拉出,置于空气中晾干1 min,再放入80 ℃烘箱中加热30 min,重复此操作2次;(c)将涂覆后的石英棒放入150 ℃烘箱中老化2 h; (d)老化后的石英棒涂层分别用10 mL丙酮、甲醇和超纯水各超声清洗10 min; (e)用刀片小心刮去多余涂层,保留涂层的长度为2.0 cm,最终得到SPME石英棒。F-COP-SPME石英棒每次使用前用10 mL甲醇和10 mL超纯水各清洗10 min后再进行萃取。

### 1.5 样品前处理

鲜活罗非鱼和基围虾购于广州当地水产品市场,将其洗净去除鱼鳞、虾皮和内脏,然后用组织匀浆机绞碎样品,放入-20 ℃下保存待分析。称取2.00 g样品放入50 mL离心管中,加入5 mL乙腈和5.00 g硫酸钠后,依次涡旋振荡和超声各10 min,再以5000 r/min速度离心10 min,移取上层清液至另一支离心管中,残渣按上述步骤重复提取一次,合并两次上清液,加入5 mL正己烷脱脂,涡旋振荡10 min,静置10 min,去除上层正己烷相,将剩余溶液在室温下氮气吹干,加3.00 mL超纯水重溶,得到样品溶液。

### 1.6 F-COP-SPME萃取过程

将3.00 mL样品溶液置于4 mL带密封垫的样品瓶中,插入制备的F-COP-SPME石英棒,涂层需全部侵入样品溶液中,室温下搅拌萃取(700 r/min) 30 min。然后将石英棒立即放入加有500 μL乙腈解吸液的小瓶中,超声解吸10 min,解吸液经0.45 μm滤膜过滤后待HPLC-UV分析。F-COP-SPME石英棒每次使用后,用10 mL甲醇和10 mL超纯水各清洗3次后待下次使用。

### 1.7 模拟计算

通过Gaussian 09和Discovery Studio软件,在密度泛函理论方法优化结构的基础上,计算丁香酚、乙酸丁香酚酯和甲基丁香酚与所制备F-COP材料间的吸附能和电子云分布情况^[20]^。

## 2 结果与讨论

### 2.1 F-COP-SPME石英棒的设计与制备

SPME涂层的制备方法主要有:物理沉积法^[17]^、溶胶-凝胶法^[21]^、化学键合法^[22]^、电化学法^[23]^、原位生长^[24]^和黏合法^[12,25]^等。其中,黏合法具有更好的通用性,适用于制备不同类型材料的SPME涂层。PAN作为一种黏合剂,除具有较好的生物相容性外,还有较高的化学和机械稳定性,适用于生物样品中活性物质的SPME^[12,25]^。F-COP结构中的F可以与丁香酚中的-OH基团形成氢键相互作用,所以F-COP对丁香酚类化合物具有特异性吸附作用。因此,本文以F-COP为吸附材料,以PAN为黏合剂制备了性能良好的F-COP-SPME石英棒,并将该石英棒用于水产品中丁香酚类麻醉剂的分析检测。制备及其应用过程见图1。

**图1:**
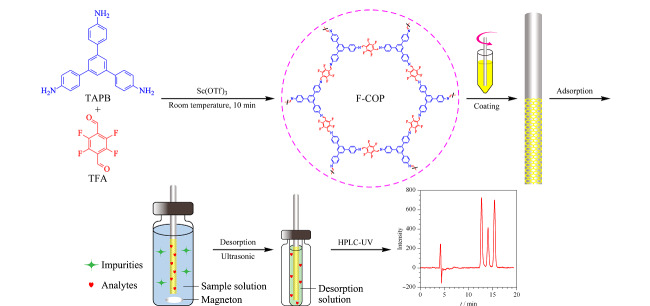
氟化共价有机聚合物固相微石英棒的制备及其应用

### 2.2 F-COP-SPME石英棒结构性能表征

通过傅里叶红外光谱(见图2a)来确定F-COP的特征官能团。从F-COP曲线可以看出,在1601 cm^-1^处存在较强吸收峰,为席夫碱反应生成的F-COP骨架中C=N的特征伸缩振动峰^[26]^,从TAPB曲线中能找到N-H(3433、3351、3209 cm^-1^)和C-N (1279 cm^-1^)的伸缩振动峰;从TFA曲线中很明显地看到1704 cm^-1^的C=O的特征吸收峰,1485 cm^-1^的C-C和1297 cm^-1^的C-F伸展振动峰。这些结果表明通过TAPB与TFA的缩合反应成功合成了F-COP材料。

**图2:**
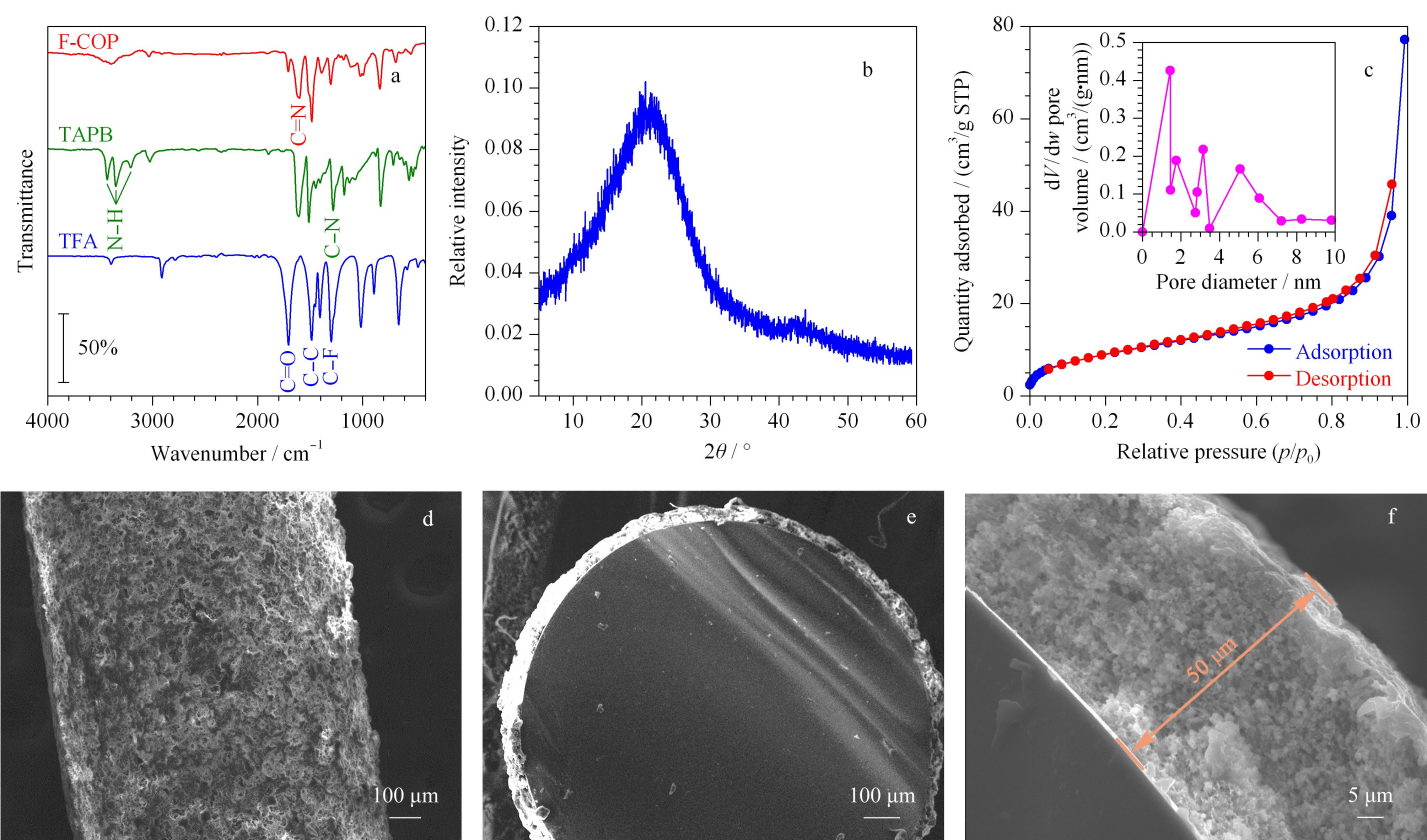
(a) F-COP、TAPB和TFA的傅里叶红外光谱图, (b) F-COP的X射线衍射图, (c) F-COP的N_2_吸附-解吸等温线和 孔径分布图, (d-f) F-COP-SPME石英棒的扫描电镜图

通过X射线衍射方法探究了F-COP的晶型结构。从图2b可以看出,F-COP的X射线衍射图中有一个宽反射峰,说明合成的F-COP是非晶态聚合物。

F-COP材料的N_2_吸附-解吸等温线如图2c所示,根据IUPAC的分类标准,属于典型的Ⅳ型,表明F-COP具有介孔特性,在低压段N_2_吸附量很快上升至较高值,说明材料中还存在微孔结构,这与孔径分布曲线(如图2c的内插图)显示的F-COP材料的平均孔径为3.69 nm的结果相一致。采用非局部密度泛函理论计算得到F-COP材料的比表面积和孔体积分别为35 m^2^/g和0.12 cm^3^/g。

通过扫描电镜对F-COP-SPME石英棒表面形貌进行表征。如图2d可见,石英棒表面粗糙、多孔,这种结构能增加涂层与样品之间的接触面积,提高样品的萃取容量。此外,从F-COP-SPME石英棒的横截面(图2e和f)看出,涂层紧密地涂覆在石英表面上,厚度约为50 μm,说明制备的石英棒牢固,可以多次重复使用。

### 2.3 F-COP-SPME石英棒的重现性和稳定性

分别取同一批次和不同批次制备的F-COP-SPME石英棒,对50 μg/L的混合标准溶液(丁香酚、乙酸丁香酚酯和甲基丁香酚)重复萃取5次,考察石英棒萃取性能的重现性。实验结果显示,同一批次和不同批次制备的F-COP-SPME石英棒之间,丁香酚、乙酸丁香酚酯和甲基丁香酚峰面积的相对标准偏差(RSD)分别小于6.3%和8.7%,表明自制F-COP-SPME石英棒具有较好的制备重现性。

将F-COP-SPME石英棒分别浸泡于水、甲醇、乙腈、丙酮、乙酸乙酯和四氢呋喃中超过24 h,均未发现涂层有溶胀和开裂的现象,表明涂层的溶剂耐受性良好。

用同一根F-COP-SPME石英棒对50 μg/L丁香酚、乙酸丁香酚酯和甲基丁香酚的混合标准溶液重复萃取10次,萃取峰面积的RSD小于4.8%,且涂层也没有明显的溶胀和脱落现象,表明自制F-COP-SPME石英棒的稳定性较好。

### 2.4 萃取条件优化

将制备的F-COP-SPME石英棒用于丁香酚类麻醉剂的萃取,考察石英棒的萃取性能。为获得最佳的萃取量,对影响萃取量的几个重要条件(萃取时间、搅拌速度、解吸溶剂及解吸时间)进行了优化。实验过程中,以萃取体积为3.00 mL,质量浓度为500 μg/L的目标物混合标准溶液考察优化条件,结果见图3。

**图3:**
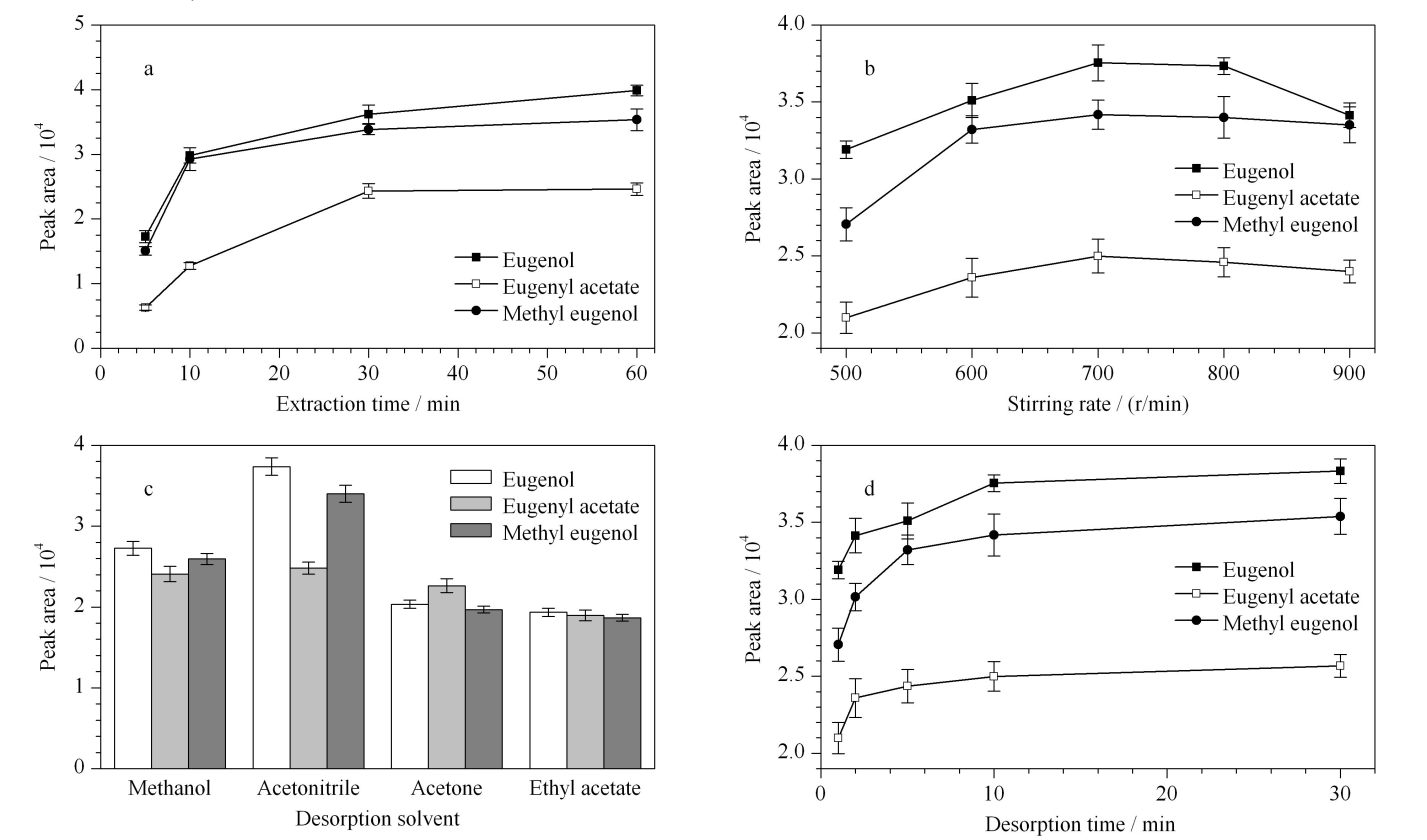
F-COP-SPME石英棒萃取条件优化(*n*=3)

2.4.1 萃取时间

SPME是一个平衡吸附过程,萃取量与萃取时间有着密切关系。实验考察了萃取时间(5~60 min)对SPME萃取量的影响。从图3a中可以看出,随着萃取时间的延长,3种麻醉剂的萃取量在30 min时基本达到了吸附平衡。随着萃取时间的进一步增加,萃取量没有明显增加。因此,为了提高样品前处理效率,选择30 min作为萃取时间。

2.4.2 搅拌速度

搅拌可以加速分析物从样品溶液向F-COP-SPME石英棒的扩散,缩短吸附平衡时间。实验中考察了不同搅拌速度下3种麻醉剂的萃取量(图3b)。实验发现,当搅拌速度达到700 r/min时,萃取率最大。继续增加搅拌速度,萃取率变化不明显,逐渐趋于平缓。所以,SPME萃取中选择搅拌速度为700 r/min。

2.4.3 解吸溶剂

解吸溶剂的选择对解吸量有很大影响,良好的解吸溶剂可以最大限度地将目标物从石英棒上洗脱下来。实验中,选择了常见的甲醇、乙腈、丙酮和乙酸乙酯溶剂作为解吸溶剂进行研究。结果如图3c所示,在相同的萃取和洗脱条件下,乙腈的解吸效果最好。因此,选择乙腈为最佳解吸溶剂。

2.4.4 解吸时间

为了实现3种麻醉剂最大程度的洗脱,实验考察了不同时间段中解吸量的变化,结果如图3d所示,3种麻醉剂的洗脱量在10 min基本达到最大值。因此,选择10 min为解吸时间。

### 2.5 吸附机理

F-COP具有富苯环和共轭双键的高交联结构,可以与丁香酚类化合物分子形成*π*-*π*堆叠作用。此外,采用分子模拟计算(Gaussian 09),探究了F-COP与丁香酚类化合物分子之间的吸附机理^[17,27]^。F-COP结构中的-F可以与丁香酚类化合物之间形成F…O-H氢键,提高目标分子的吸附能力,F-COP的这些特性使其成为优良的SPME介质。分子模拟发现,丁香酚、乙酸丁香酚酯和甲基丁香酚均稳定地存在于F-COP空腔结构中(见图4)。从图4a可知,丁香酚可以与F-COP骨架结构中的N和F形成氢键,氢键键长分别为0.191和0.232 nm,两种氢键的共同作用,使得F-COP与丁香酚之间有较强的吸附,其吸附能为49.9 kJ/mol。相比于丁香酚,F-COP与乙酸丁香酚酯和甲基丁香酚之间不存在氢键相互作用,因此吸附较弱,其吸附能分别为17.9和19.4 kJ/mol。在此基础上,利用Discovery Studio软件模拟计算了F-COP与丁香酚类化合物分子之间的电子云分布。从图4d和f可知,F-COP与丁香酚和甲基丁香酚之间存在电荷转移,因此主客体分子间吸附较强。从图4e可知,F-COP与乙酸丁香酚酯之间几乎不存在电荷转移,因此主客体分子间吸附较弱。上述的模拟计算结果与实验结果吻合,即F-COP与丁香酚之间识别能力最高,甲基丁香酚次之,乙酸丁香酚酯最弱。

**图4:**
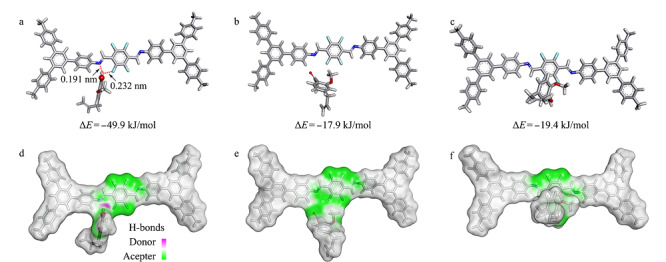
F-COP与目标分子间吸附能力及电子云分布理论模拟图

### 2.6 方法的验证

在优化的萃取条件下,探究了3种丁香酚类麻醉剂的线性关系、线性范围、相关系数(*r*^2^)及检出限(LOD)等性能参数。结果如表1所示,丁香酚和乙酸丁香酚酯在10~1000 μg/L,甲基丁香酚在10~1500 μg/L范围内具有良好的线性关系,*r*^2^大于0.9961。3种丁香酚类麻醉剂的LOD(*S/N*=3)在2.9~4.5 μg/kg之间,该法的检出限可以满足日本和欧盟等国家对丁香酚类麻醉剂在水产品体内残留最高限量为50 μg/kg的检测要求。

**表1:** F-COP-SPME-HPLC-UV检测3种丁香酚类麻醉剂的方法性能参数

Analyte	Linear equation	Linear range/ (μg/L)	Correlation coefficient (r^2^)	LOD/ (μg/kg)	RSDs/%^a)^	
					Intra-batch	Inter-batch
Eugenol	Y=6.48×10^4^X+7.6×10^2^	10-1000	0.9974	2.9	4.4	6.7
Eugenyl acetate	Y=4.17×10^4^X+7.7×10^2^	10-1000	0.9961	4.5	6.3	8.7
Methyl eugenol	Y=5.77×10^4^X+9.8×10^2^	10-1500	0.9963	3.3	5.8	7.3

*Y*: peak area; *X*: mass concentration, μg/L. a). Calculated at the mass concentration level of 50 μg/L (*n*=5).

与文献报道的水产品中丁香酚类麻醉剂的检测方法相比,本研究建立的方法的检出限较低,可以满足水产品中丁香酚、乙酸丁香酚酯和甲基丁香酚3种麻醉剂残留的同时检测。几种方法的线性范围和检出限见表2。

**表2:** 本方法与文献报道的水产品中丁香酚类麻醉剂检测方法比较

Method	Samples	Linear range/ (μg/L)		LODs/(μg/kg)			Ref.
				Eugenol	Eugenyl acetate	Methyl eugenol	
SPE-GC-MS	fish	5-	500	0.4	-	0.2	[4]
DSPE-HPLC-MS	shrimp, crab, carp	5-	500	1.47	-	-	[8]
LLE-HPLC-UV	tilapia	100-	10000	30	-	-	[9]
MISPE-HPLC-UV	grouper, prawn	50-	10000	15	-	15	[10]
F-COP-SPME-HPLC-UV	tilapia, shrimp	10-	1000	2.9	4.5	3.3	this work

DSPE: dispersive SPE; LLE: liquid-liquid extration; MISPE: molecularly imprinted solid phase extraction. -: not detected.

### 2.7 实际样品分析及加标回收率试验

采用所建立的F-COP-SPME-HPLC-UV方法对罗非鱼和基围虾样品进行分析检测,在罗非鱼样品中检测出丁香酚含量为101 μg/kg,超出了日本和欧盟等国家对丁香酚类麻醉剂在水产品体内残留限量为50 μg/kg的标准。实际样品的相关谱图见图5。

**图5:**
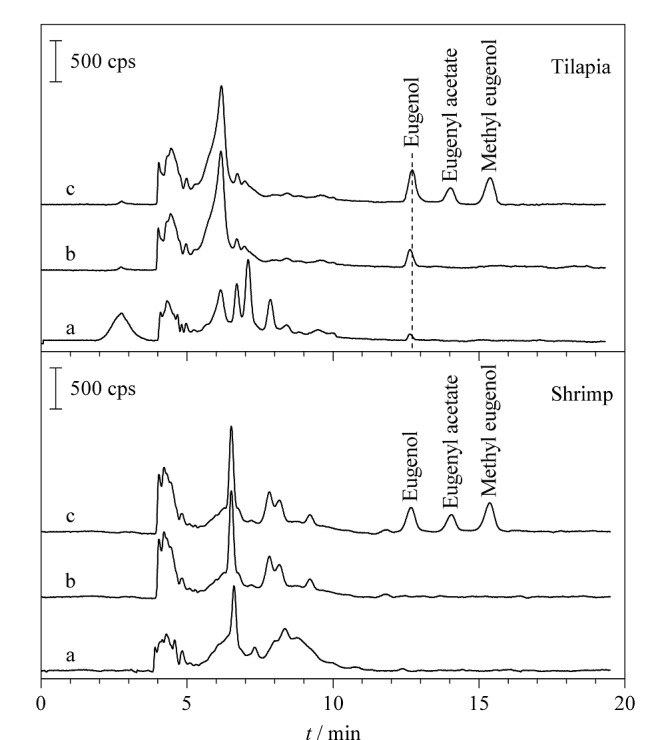
罗非鱼和基围虾样品的HPLC-UV图

采用在实际样品中加标验证方法的可靠性,加标水平分别为50和100 μg/kg。3种麻醉剂在两种样品中的加标回收率分别为76.7%~98.7%和80.3%~104%, RSD分别为8.5%~11.8%和8.6%~12.4%(见表3),表明所建立方法具有良好的精密度和准确度,可以满足实际水产品中丁香酚、乙酸丁香酚酯和甲基丁香酚类麻醉剂残留的分析要求。

**表3:** 3种丁香酚类麻醉剂在水产品中的加标回收率及RSD(*n*=5)

Sample	Analyte	Found/ (μg/kg)	Spiked levels				
			50 μg/kg			100 μg/kg	
Rec./ %	RSD/ %	Rec./ %	RSD/ %
Tilapia	eugenol	101	76.7	10.6		80.1	8.5
	eugenyl acetate	ND	81.2	11.8		98.7	11.2
	methyl eugenol	ND	87.9	11.5		92.4	10.2
Shrimp	eugenol	ND	80.3	9.4		86.6	8.6
	eugenyl acetate	ND	97.7	11.5		104	11.3
	methyl eugenol	ND	98.9	12.4		94.7	10.9

Rec.: recovery. ND: not detected or lower than the LOD.

## 3 结论

本研究制备了新型氟化共价有机聚合物,将其作为SPME石英棒涂层吸附剂,制备了F-COP-SPME石英棒,结合HPLC-UV建立了测定丁香酚、乙酸丁香酚酯和甲基丁香酚的分析方法,并将该方法成功地应用到水产品中丁香酚类麻醉剂的残留检测中,方法操作简单,线性范围宽,检出限低,适用于水产品中丁香酚、乙酸丁香酚酯和甲基丁香酚3种麻醉剂残留的同时分析。
